# Hundreds of Nurses Join Its Register

**Published:** 1918-01-12

**Authors:** 


### THE COLLEGE OF NURSING

**(Limited by Guarantee).**

### HUNDREDS OF NURSES JOIN ITS REGISTER.

It is of the first importance that we are in a position this week to give incontrovertible and cheering evidence that trained nurses throughout the country are resolved to do their utmost to help themselves and their profession by becoming at once members of the College and joining its Register in ever-increasing numbers. The Bolos have exhibited ill-judgment by the methods they have pursued, especially of late, which demonstrate the contempt in which they hold the intelligence of trained nurses in this country. It was time that trained nurses should assert themselves. The subjoined lists demonstrate that this view is now taken by trained nurses everywhere, for they include the training-schools of English, Welsh, Scotch, Irish, Isle of Man, New South Wales, and South African institutions, as well as of great Poor-Law institutions in London, the provinces, and Scotland. A careful study of the names of the schools enumerated, with the help of "Burdett's Hospitals and Charities," 1917, will well repay the time thus spent by members of Parliament, supporters of our hospitals, training-schools and Poor-Law institutions, matrons, sisters, and nurses. The lists are a splendid demonstration that the College of Nursing is making steady and increasing progress. Further, the public, it is fair to conclude, who study these lists, will gratefully and continuously lend their eleemosynary aid to the Building and Scholarship Funds now being raised by the British Women's Hospital Committee. Providing trained nurses continue to make it their business to register with the College without delay, and to take part in the election of members of its Council next April, the nurses may themselves win State Registration with practical certainty before the year is out.

### LIST NO. 2 OF TRAINING-SCHOOLS OF REGISTERED NURSES.

At the meeting of the Council of the College of Nursing held on December 6, 1917, the candidates passed for membership of the College and the College Register were trained at the following training-schools:—

Guy's Hospital, London; King's College Hospital, S.E.; Hampstead General and North West London Hospital; University College Hospital, S.E.; London Hospital, Whitechapel, E.; Middlesex Hospital, London; Mildmay Mission Hospital, Bethnal Green; St. Thomas's Hospital, London; Prince of Wales's General Hospital, N.; St. Bartholomew's Hospital, E.C.; St. Mary's Infirmary, Highgate, N.; Camberwell Infirmary, S.E.; St. James' Infirmary, Wandsworth, S.W.; Southwark Infirmary, East Dulwich Grove, S.E.; Lambeth Infirmary, S.E.; Princess Christian Hospital, Weymouth; Cumberland Infirmary, Carlisle; Royal Gwent Hospital, Newport; Royal County Hospital, Guildford; Royal South Hants and Southampton Hospital; Royal Hospital, Sheffield; Royal Devon and Exeter Hospital, Exeter; Royal Infirmary, Halifax; Royal Hospital, Richmond, Surrey; Addenbrooke's Hospital, Cambridge; Royal Infirmary, Sunderland; General Infirmary, Chester; Ancoats Hospital, Manchester; Royal Infirmary, Manchester; General Hospital, Nottingham; County Hospital, Lincoln; Royal Victoria Hospital, Folkestone; Royal Albert Edward Infirmary and Dispensary, Wigan; Royal Berkshire Hospital, Reading; Royal Sussex County Hospital, Brighton; District Infirmary, Ashton-under-Lyne; Norfolk and Norwich Hospital, Norwich; General Hospital, Bristol; Royal Infirmary, Bristol; Wolstanton and Burslem Poor-Law Institution; Tonbridge Poor-Law Institution, Tunbridge Wells; Croydon Infirmary; Ashton-under-Lyne Poor-Law Institution; Aston Union Infirmary; Bolton Poor-Law Institution (Townley's Hospital); Mill Road Infirmary, Liverpool; Oldham Poor-Law Institution; North Bierley Union Infirmary, Clayton; Brownlow Hill Poor-Law Institution, Liverpool; St. Vincent's Hospital, Darlinghurst, Sydney, N.S.W.; Seamen's Hospital, Greenwich, with New Hospital for Women, Soho Square, London; New Hospital for Women with Seamen's Hospital, Greenwich; Dr. Steevens' Hospital, Dublin; Royal Victoria Hospital, Belfast; Mater Infirmorum Hospital, Belfast; Barrington's Hospital, Limerick; South Infirmary, Cork; Clare County Infirmary; Belfast Union Infirmary; and Royal Infirmary, Sheffield.

### LIST NO. 3 OF TRAINING-SCHOOLS OF REGISTERED NURSES.

At the meeting of the Council of the College of Nursing held on January 3, 1918, the candidates passed for membership of the College and the College Register were trained at the following training-schools:—

St. Bartholomew's Hospital, London; St. George's Hospital, S.W.; King's College Hospital, London; London Hospital, Whitechapel; Middlesex Hospital, London; St. Thomas's Hospital, London; University College Hospital, London; Westminster Hospital, S.W.; Guy's Hospital, London; London Temperance Hospital, N.W.; Royal Free Hospital, W.C.; St. Mary's Hospital, Paddington; West London Hospital, W.; Bethnal Green Infirmary, N.E.; St. Luke's Infirmary, Chelsea, S.W.; Fulham Infirmary, London; Holborn Infirmary, Highgate; St. George's Infirmary, Fulham Road; St. George-in-the-East Infirmary; St. Mary, Islington, Infirmary, N.; St. Pancras Infirmary, N.; West Ham Infirmary, Leytonstone; Willesden Infirmary, N.W.; North Devon Infirmary, Barnstaple; Royal Berkshire Hospital, Reading; General Hospital, Birmingham; Queen's Hospital, Birmingham; Royal Infirmary, Bradford; City of London Infirmary, Homerton, N.E.; St. Pancras Infirmary, South, London; Lambeth Infirmary, S.E.; Suffolk General Hospital, Bury St. Edmunds; Royal Infirmary, Bristol; General Hospital, Bristol; General Infirmary, Burton-on-Trent; King Edward VII.'s Hospital, Cardiff; General Hospital, Cheltenham; Chesterfield and North Derbyshire Hospital; General Hospital, Darlington; Royal Infirmary, Derby; Royal Albert Hospital, Devonport; Royal Infirmary, Doncaster; Royal Victoria Hospital, Folkestone; General Infirmary, Gloucester; Royal Infirmary, Halifax; West Herts Hospital, Hemel Hempstead; County Hospital, Hertford; General Infirmary, Leeds; Royal Infirmary, Leicester; David Lewis Northern Hospital, Liverpool; Royal Infirmary, Manchester; General Hospital, Nottingham; Royal Isle of Wight County Hospital, Ryde; Royal Infirmary, Sheffield; Royal Salop Infirmary, Shrewsbury; Stockport Infirmary; North Staffordshire Infirmary and Eye Hospital, Stoke-on-Trent; Warrington Infirmary and Dispensary; Princess Christian Hospital, Weymouth; General Hospital, Gt. Yarmouth; Ashton-under-Lyne Poor-Law Institution; Higher Tranmere Poor-Law Institution, Birkenhead; North Bierley Union Infirmary, Clayton, Bradford; Medway Union Infirmary, Chatham; Farnham Poor-Law Institution; Anlaby Road Infirmary, Kingston-upon-Hull; Leeds Infirmary (Union); Bagthorpe Infirmary, Nottingham; Ecclesall Bierlow Poor

Law Institution, Sheffield; Fir Vale Poor-Law Hospital, Sheffield; Crumpsall Poor-Law Institution, Manchester; Warwick Poor-Law Institution; North-West London Hospital, N.W.; St. Marylebone Infirmary, Notting Hill; Whitechapel Infirmary, E.; St. Saviour's Infirmary, South wark; Royal Southern Hospital, Liverpool; South Staffordshire General Hospital, Wolverhampton; Victoria Hospital, Burnley; Kent and Canterbury Hospital, Canterbury; East Sussex Hospital, Hastings; Royal Hospital, Portsmouth; General Hospital, Salisbury; Middlesbrough Poor-Law Institution; North Evington Poor-Law Institution, Leicester; Dudley Road Infirmary, Birmingham; Bradford Poor-Law Institution; Burnley Poor-Law Institution; Kingston-upon-Hull Infirmary; Highfield Infirmary, Knotty Ash, Liverpool; Mill Road Infirmary, Liverpool; Salford Infirmary, Pendleton, Manchester; Harton Poor-Law Institution, South Shields; Steyning Poor-Law Institution; Wolstanton and Burslem Poor-Law Institution; Carnarvon Hospital, Kimberley; Brompton Hospital, S.W., and St. Mary's Hospital, W.; Seamen's Hospital, Greenwich, S.E., and New Hospital for Women, N.W.; Kensington Infirmary; Toxteth Park Infirmary, Liverpool; Royal Hants County Hospital, Southampton; Western Infirmary, Glasgow; Royal Infirmary, Edinburgh; Royal Infirmary, Glasgow; Royal Infirmary, Dundee; Dumfries and Galloway Royal Infirmary; Royal Infirmary, Aberdeen; County Hospital, Ayr; Town's Hospital, Glasgow; Stobhill Hospital, Glasgow; Kilmarnock Infirmary; Western District Hospital, Glasgow; Merryflatts Hospital, Govan; Barnhill Hospital, Glasgow; Belvidere Fever Hospital, Glasgow; Royal Infirmary, Perth; Hospital for Sick Children, Great Ormond Street, London; Sir Patrick Dun's Hospital, Dublin; Chelsea Infirmary, S.W.; County Hospital, Durham; Royal Devon and Exeter Hospital, Exeter; Royal Surrey County Hospital, Guildford; Gloucestershire Royal Infirmary and Eye Institution; Royal Infirmary, Hull; Herefordshire General Hospital, Hereford; Noble's Isle of Man General Hospital; Victoria Central Hospital, Liscard; County Hospital, Lincoln; West Kent General Hospital Maidstone; Ancoats Hospital, Manchester; Royal Infirmary, Oldham; South Devon and East Cornwall Hospital, Plymouth; Royal Portsmouth, Portsea, and Gosport Hospital, Portsmouth; Royal Hospital, Salford; General Infirmary, Salisbury; Royal Hospital, Sheffield; Staffordshire General Infirmary, Stafford; Stockton and Thornaby Hospital; General and Eye Hospital, Swansea; Clayton Hospital, Wakefield, Yorks; General Infirmary, Worcester; Aston Poor-Law Institution, Birmingham; Erdington Poor-Law Institution, Birmingham; Bolton Poor-Law Institution (Townley's Hospital); Bromley Poor-Law Institution; Township of South Manchester Hospitals, West Didsbury; Warrington Poor-Law Institution; Swansea Poor-Law Institution; Stepping Hill Poor-Law Hospital, Stockport; North Ormesby Hospital, Middlesbrough, Yorks; Provincial Hospital, Port Elizabeth, South Africa; National Hospital, Queen Square, and Essex County Hospital, Colchester; National Hospital, Queen Square, and St. Mary's Infirmary, Islington; Brownlow Hill Infirmary, Liverpool.

